# Relapse of COVID-19 and Viral Evolution in a Patient With Good Syndrome: A Case Report

**DOI:** 10.7759/cureus.52592

**Published:** 2024-01-19

**Authors:** Mika Iwasaki, Masao Hashimoto, Junko S Takeuchi, Yusaku Kusaba, Moto Kimura, Junko Terada-Hirashima, Wataru Sugiura, Masayuki Hojo

**Affiliations:** 1 Department of Respiratory Medicine, National Center for Global Health and Medicine, Tokyo, JPN; 2 Center for Clinical Sciences, National Center for Global Health and Medicine, Tokyo, JPN

**Keywords:** good syndrome, viral evolution, relapse, covid-19, sars-cov-2

## Abstract

Delays in clearance and rapid evolution of severe acute respiratory syndrome coronavirus 2 (SARS-CoV-2) have been reported in immunocompromised patients. We encountered a case of recurrent, multi-mutational SARS-CoV-2 infection in a 40-year-old man with severe immunodeficiency due to Good syndrome. The patient had not received the SARS-CoV-2 vaccination. In August 2021, he was first admitted to the hospital owing to coronavirus disease 2019 (COVID-19) pneumonia and was administered dexamethasone, remdesivir, and baricitinib. Although his fever and respiratory condition improved once, chest computed tomography (CT) revealed extensive diffuse consolidation and ground-glass opacities (GGOs), and both methylprednisolone pulse therapy and tocilizumab yielded a limited effect. After a third course of remdesivir without immunosuppressants or steroids, the patient recovered, and he tested negative for SARS-CoV-2. On day 272 since the clinical onset, he was readmitted with dyspnea and mild fever due to a COVID-19 recurrence. He was infected with the Delta variant (AY.29), despite the Omicron (BA.2) variant being predominant at that time. During this admission, additional remdesivir and casirivimab/imdevimab yielded marked effects, and the SARS-CoV-2 quantitative reverse transcriptase-polymerase chain reaction (qRT-PCR) tests rapidly returned negative. Phylogenetic analysis demonstrated the accumulation of mutations, including those yielding remdesivir resistance, throughout the SARS-CoV-2 genome. Appropriate use of antivirals and monoclonal antibodies may aid in the recovery of patients with COVID-19 and immunodeficiency and in preventing the emergence of multi-mutational SARS-CoV-2 variants.

## Introduction

Immunocompromised conditions expose patients to an increased risk of severe forms of coronavirus disease 2019 (COVID-19) pneumonia [1]. Delayed viral clearance and rapid virus evolution have been observed in immunocompromised patients, but no relevant treatment guidelines have been established [2]. Good syndrome is a rare adult-onset primary immunodeficiency characterized by thymoma and hypogammaglobulinemia.

We report a case of recurrent severe acute respiratory syndrome coronavirus 2 (SARS-CoV-2) infection in a patient with severe immunodeficiency due to Good syndrome.

## Case presentation

On August 26, 2021, a 40-year-old male was admitted to the hospital due to a cough and fever persisting since August 11 (day 0). The patient had a history of type 1 diabetes and Masaoka stage I thymoma with hypogammaglobulinemia (Good syndrome, diagnosed two years ago based on peripheral blood investigations demonstrating hypogammaglobulinemia and flow cytometry of bone marrow aspirates, which revealed highly reduced B-cells). The patient was only treated symptomatically and did not receive any SARS-CoV-2 vaccination. He required 1 L of oxygen per minute via a nasal cannula, and chest computed tomography (CT) revealed ground-glass opacities (GGOs) in both lungs and an anterior mediastinal tumor (data not available). The patient's diagnosis of severe COVID-19 accompanied by pneumonia was confirmed by a positive result of the quantitative reverse transcriptase-polymerase chain reaction (qRT-PCR) of his nasopharyngeal swab sample for SARS-CoV-2. The patient was administered dexamethasone 6 mg orally, remdesivir intravenously, and baricitinib orally for 10, five, and five days, respectively. Although qRT-PCR was not performed, the patient’s fever and respiratory condition improved, and he was discharged on day 28.

**Figure 1:**
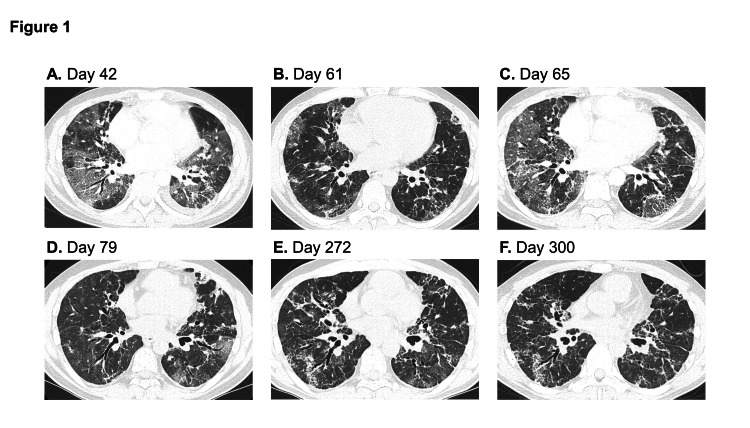
A series of chest computed tomography (CT) images of the patient with Good syndrome with recurrent COVID-19. Denotes the number of days after symptom onset. A. On day 42, extensive ground-glass opacities (GGOs) and consolidation were observed in both lungs. B. On day 61, the GGOs and consolidation improved after the administration of remdesivir, corticosteroids, and tocilizumab. C. On day 65, new GGOs emerged bilaterally. D. On day 79, only a few patchy GGOs remained at the time of discharge. E. On day 272, various abnormal shadows, such as reticulonodular shadows and bronchiectasis, were observed. Moreover, new consolidations and GGOs emerged. F. On day 300, the patient’s respiratory condition improved, and most GGOs had turned into reticular shadows.

However, nine days after discharge, he was admitted again due to a recurrence of fever and cough. Dexamethasone (6 mg) was restarted; however, his respiratory condition worsened. The patient was transferred to our hospital on day 42 for further treatment. Chest CT showed extensive diffuse consolidation and GGOs (Figure 1A).

The SARS-CoV-2 qRT-PCR test result was positive. The patient was administered methylprednisolone 1,000 mg intravenously for three consecutive days, remdesivir for five consecutive days, and tocilizumab 440 mg intravenously once. His respiratory condition and GGOs on chest CT showed improvement (Figure 1B); therefore, the corticosteroid dose was tapered. However, his respiratory condition gradually deteriorated, and new GGOs emerged in subsequent CTs (Figure 1C). The SARS-CoV-2 qRT-PCR test on day 66 remained positive, but there were no other illnesses. We suspected severe immunodeficiency that contributed to delayed clearance of SARS-CoV-2 and inhibited the intensification of anti-inflammatory therapy. Remdesivir was restarted without immunosuppressants or steroids, and his respiratory condition and GGO levels improved (Figure 1D). After discharge, the patient received regular immunoglobulin replacement therapy. During his follow-up visit, the SARS-CoV-2 qRT-PCR test result was negative.

On day 272, the patient returned to our hospital (third hospitalization) with dyspnea and mild fever (Figure 1E). The SARS-CoV-2 qRT-PCR test results were positive. Given his immunodeficiency, the patient received additional remdesivir and casirivimab/imdevimab. His respiratory condition improved (Figure 1F), and the SARS-CoV-2 qRT-PCR test quickly turned negative.

**Figure 2:**
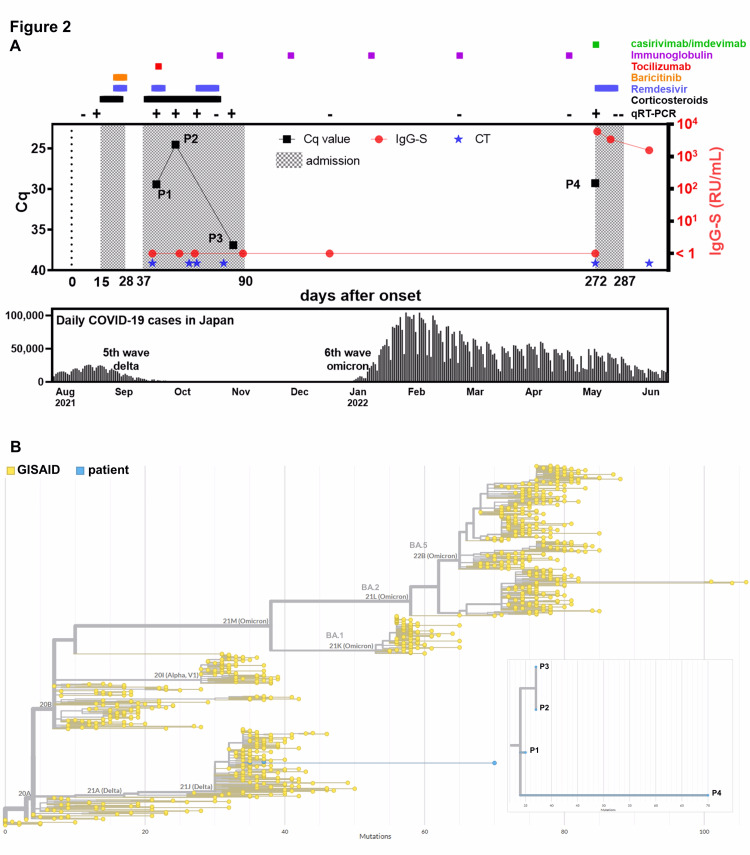
The clinical course of the 40-year-old male patient with recurrent SARS-CoV-2 infection. A. The upper panel shows the clinical course of the patient. Black squares, red circles, and blue stars indicate Cq values, IgG-S, and CT scans, respectively. SARS-CoV-2 positivity (qRT-PCR) is indicated in the upper part of the graph by a + (positive) or – (negative) sign. Each treatment regimen is indicated in the upper part of the graph by horizontal lines. The bottom panel represents the number of daily new cases of SARS-CoV-2 infections in Japan. B. Phylogenetic analysis was performed using patient-derived sequences (blue circle, n = 4) and sequences derived from the GISAID database (yellow circle, n = 778). The branch length corresponds to nucleotide divergence. A cluster of patient-derived sequences is enlarged in the lower right frame. Cq: quantification of cycle; GISAID: Global Initiative on Sharing All Influenza Data

Serological assays showed that the immunoglobulin G antibodies against SARS-CoV-2 spike protein (IgG-S) were undetectable at six time points from day 42 to day 272 (Figure 2A).

On day 273 (the day after the initiation of casirivimab/imdevimab administration), IgG-S turned positive and decayed over time but remained positive until the end. The IgG-S positivity after day 273 could have been derived from casirivimab/imdevimab, not from the patient-derived antibodies. The patient tested negative for IgG-N throughout the observation period, suggesting that the patient could not produce neutralizing antibodies (Appendix A).

We also performed quantitative SARS-CoV-2 viral load measurements and whole-genome sequencing at four time points using nasopharyngeal swab samples. The viral load peaked on day 54 (P2, quantification of cycle (Cq) = 24.5) after the COVID-19 diagnosis after the COVID-19 diagnosis. After testing negative on day 90, it tested positive again on day 272 (P4; Cq = 29.3). All patient-derived viruses (P1-P4) were classified as Delta variants (AY.29). Notably, the Delta variant was prevalent in Japan at the time of the patient’s second admission, whereas at the time of the third admission, the Omicron (BA.2) variant was predominant [3, 4]. These observations support the fact that the patient was not reinfected with different viruses but had a recurrent infection. We performed a phylogenetic analysis along with control sequences from the Global Initiative on Sharing All Influenza Data (GISAID) database. All patient-derived sequences were tightly clustered together in the tree, but the sequence on day 272 had a longer branch length, indicating that multiple mutations had accumulated in the genome (Figure 2B).

## Discussion

We treated a patient with recurrent SARS-CoV-2 infection, wherein multiple virus mutations had accumulated due to severe immunodeficiency resulting from Good syndrome. Primary immunodeficiency is a risk factor for severe COVID-19 [1]. Good syndrome is a type of primary immunodeficiency associated with thymoma and hypogammaglobulinemia, accounting for 0.6%-5% of all thymomas [5]. To date, only eight cases of Good syndrome associated with COVID-19 have been reported [6]. This is the first report in which the viral genome has been investigated in detail.

In this case, the treatment course was characterized by two factors. First, immunosuppressive therapy, such as corticosteroids, did not improve the patient’s condition, as this patient was incapable of producing neutralizing antibodies due to Good syndrome. Lack of humoral immunity may delay viral clearance, leading to COVID-19 recurrence [7]. Furthermore, corticosteroids may prolong the duration of viral shedding [8]. Therefore, we did not administer corticosteroids or immunosuppressants during the third round of hospitalization, and he was discharged after 15 days of readmission. Antiviral and antibody-based therapies should be considered rather than corticosteroids or immunosuppressants in immunocompromised patients. Second, the disease improved with antiviral drugs and monoclonal antibodies without increasing the corticosteroid dose. Remdesivir has shown efficacy against mild to severe COVID-19, including in immunosuppressed patients [9, 10]. However, viral resistance to remdesivir has been reported in vivo [11]. In contrast, monoclonal antibodies are effective only in patients who do not require oxygen administration [12]. However, the efficacy of monoclonal antibodies was reported in hospitalized patients who tested negative for anti-SARS-CoV-2 antibodies, suggesting that their use at the time of third hospitalization in this case may have led to rapid improvement in the condition [13]. Since it may be difficult to acquire antibodies through vaccination, administration of monoclonal antibodies in advance, according to the prevalent strain, may be an option to prevent relapse and re-infection in immunocompromised patients. Although monoclonal antibodies are effective against the delta variants, as reported, the efficacy of existing monoclonal antibodies decreases after the emergence of the omicron variants [14]. Therefore, the development of monoclonal antibodies against new mutant strains and effective convalescent plasma therapy are strongly recommended.

**Figure 3:**
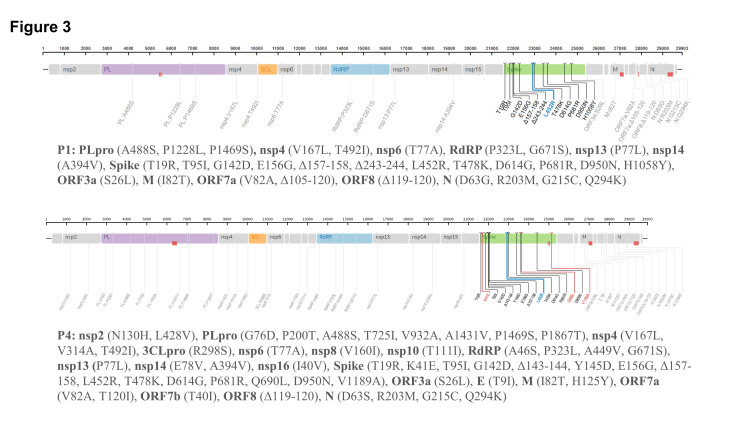
Whole genome sequencing of patient-derived SARS-CoV-2 Schematic representation of the locations of amino acid mutations in the patient at two sampling time points, P1 (upper) and P4 (lower), as compared with Wuhan-Hu-1 (NC_045512.2). The images were generated by the Stanford SARS-CoV-2 Sequence Analysis tool (https://covdb.stanford.edu/sierra/sars2/by-sequences/). Red-shaded areas are positions that were not sequenced or aligned.

Interestingly, whole-genome sequencing confirmed the acquisition of A449V in the RNA-dependent RNA polymerase (RdRp) gene on day 272 (Figure 3).

This mutation is reportedly associated with remdesivir resistance [15]. Further studies are warranted to study the replication efficiency and remdesivir susceptibility of the virus. Next, casirivimab/imdevimab treatment was not initiated during the observation period for whole-genome viral sequencing. Nevertheless, we observed multiple mutations in the S gene, including a convergent mutation (143-144 deletions), which has been independently reported in immunocompromised patients [16]. Deletions between residues 141 and 146 occurred in some variants of concern (ex. alpha and omicron BA.1), and they were associated with resistance to several monoclonal antibodies targeting the N-terminal domain of the S protein (although casirivimab/imdevimab target the receptor-binding domain).

## Conclusions

We present a case of COVID-19 associated with Good syndrome; improvement was noted with the use of antivirals and monoclonal antibodies. Within-host accelerated evolution of SARS-CoV-2 was observed during persistent infections and relapses. It is important to investigate the longitudinal changes in viral genome sequencing, be aware of the emergence of novel variants, and select treatment modalities accordingly.

## Appendices

Appendix A

Supplementary Methods

Serological assays: Using residual serum samples, we performed two serological assays to detect the immunoglobulin G (IgG) antibodies against SARS-CoV-2 spike and nucleocapsid proteins (IgG-S and IgG-N, respectively). To detect IgG-S, the EUROIMMUN Anti-SARS-CoV-2 QuantiVac ELISA (IgG) (Euroimmun, Germany) was used, according to the manufacturer’s protocol. We considered ≥ 11 RU/mL as the cutoff for seropositivity, as recommended by the vendor. To detect IgG-N, the EUROIMMUN Anti-SARS-CoV-2-NCP ELISA (IgG) (Euroimmun) was used, in accordance with the manufacturer’s instructions. The results were calculated as the ratio of the extinction of the control or sample to that of the calibrator. We defined a ratio of ≥ 1.1 as seropositive, according to the vendor’s recommendation. Each serum sample was tested in duplicate.

Viral loads: SARS-CoV-2 quantitative reverse transcriptase-polymerase chain reaction (qRT-PCR) tests were performed using the SARS-CoV-2 Detection Kit-Multi (Toyobo, Japan) and LightCycler 96 System (Roche, Switzerland), in accordance with the manufacturer’s instructions. Briefly, the cycle quantification (Cq) values were obtained by amplifying two regions (N1 and N2) derived from the N gene. Each sample was tested in duplicate, and the mean Cq values of the N1 set are shown in Figure 2A.

Virus whole-genome sequencing and phylogenetic analysis: Total RNA was extracted from nasopharyngeal swab samples, followed by cDNA synthesis, target amplification, library preparation using Illumina COVIDseq with ARTIC V4.1, and SARS-CoV-2 genome sequencing on Illumina iseq 100. The resulting sequences were applied to the NextStrain pipeline for phylogenetic analysis (NextStrain cli 6.1.0. post1 with a build for ncov-v12 (https://github.com/nextstrain/ncov)). Random worldwide viral genome sequences (n = 778), including two reference genomes (MN908947.3 and OM131541.1), were obtained from GISAID (https://gisaid.org/) as controls.
